# Gender differences in the use of atypical antipsychotics in early-onset schizophrenia: a nationwide population-based study in Brazil

**DOI:** 10.1186/s12888-021-03327-7

**Published:** 2021-06-29

**Authors:** Izabela Fulone, Marcus Tolentino Silva, Luciane Cruz Lopes

**Affiliations:** Pharmaceutical Sciences Graduate Course, University of Sorocaba, UNISO, Sorocaba/State of São Paulo, Brazil

**Keywords:** Schizophrenia, Child, Adolescent, Antipsychotic agents, Gender difference

## Abstract

**Background:**

The use of atypical antipsychotics for the treatment of schizophrenia and other mental disorders in populations under 18 years of age is increasing worldwide. Little is known about treatment patterns and the influence of gender differences, which may be a predictor of clinical outcomes. The aim of this study was to investigate gender differences in the use of atypical antipsychotics in patients with early-onset schizophrenia (EOS) assisted by the public health system in Brazil.

**Methods:**

We conducted a cross-sectional study of outpatients with EOS aged 10 to 17 years who received at least one provision of atypical antipsychotics (clozapine, olanzapine, risperidone, quetiapine or ziprasidone) from a large Brazilian pharmaceutical assistance programme. Data were retrieved from a nationwide administrative database from 2008 to 2017.

**Results:**

Of the 49,943 patients with EOS, 63.5% were males, and the mean age was 13.6 years old. The patients were using risperidone (62.5%), olanzapine (19.6%), quetiapine (12.4%), ziprasidone (3.3%) and clozapine (2.2%). We found gender differences, especially in the 13–17 year age group (65.1% for males vs. 34.9% for females, *p* < 0.001), in the use of risperidone (72.1% for males vs. 27.9% for females, *p* < 0.001) and olanzapine (66.5% for males vs. 33.5% for females, *p* < 0.001). Only in the 13 to 17 years age group were the prescribed doses of olanzapine (*p* = 0.012) and quetiapine (*p* = 0.041) slightly higher for males than for females.

**Conclusions:**

Our findings showed gender differences among patients diagnosed with EOS and who received atypical antipsychotics. More attention should be devoted to gender differences in research and clinical practice.

## Background

Early-onset schizophrenia is defined as schizophrenia diagnosed before 18 years old. The differential diagnosis is difficult, and approximately 30 to 50% of patients with affective or other atypical psychotic symptoms are misdiagnosed with early-onset schizophrenia [1].

The prevalence of psychotic disorders in the 10 to 18 years age group is relatively low (approximately 0.4%) [2]. The onset of schizophrenia prior to age 13 years is rarer, affecting 1.6 to 1.9 per 100,000 child population, but between the ages of 13 and 17 years old, the prevalence increases more rapidly [3].

The frequency and duration of psychotic episodes could have deleterious neuropsychological, neurophysiological, and neurostructural effects, particularly in children and adolescents [1]. For this reason, it is important to optimize early diagnosis and start an appropriate treatment to improve outcomes [4].

Many studies have reported gender differences, which highlights the association between male gender and earlier age of onset schizophrenia [2, 5, 6]. Male schizophrenia patients tend to show an earlier age of onset, more negative symptoms, higher relapse rate, poor outcomes and worse responses to antipsychotics than female schizophrenic patients [6]. Other studies have not endorsed the gender difference and showed controversial results or no significant differences between males and females [4, 7, 8].

Pharmacological treatment in this age group is challenging from both a clinical and ethical perspective [9]. There are few studies and limited data based on evidence that supports the effectiveness, safety, and effects of long-term antipsychotic (typic and atypical) use in children and adolescents, as it is a rare disorder and is difficult to conduct trials in this group age [10]. In recent years, there has been no significant increase in the number of trials addressing it with this particular age group [11].

However, antipsychotics have long been seen as playing a key role in the treatment of schizophrenia in children and adolescents [9, 12]. There is no convincing evidence about the superiority of atypical antipsychotics over typical antipsychotics, but atypical antipsychotics have been considered the first line of pharmacological treatment in EOS due to the lower incidence of adverse reactions and extrapyramidal symptoms and are more acceptable than typical antipsychotics [10].

Most of the recommendations shown in the guidelines for the treatment of schizophrenia in children and adolescents are extrapolations of existing guidelines for adults due to the lack of specific evidence about this age range [9, 13, 14]. Many antipsychotics are not product licenced for use in children and/or adolescents, and prescribers and parents should be aware and responsible for their use [9, 15, 16].

However, studies have reported an increase in the use of antipsychotics in this group age and an increase in the incidence of early-onset schizophrenia and other mental disorders in recent years [4, 8, 17]. Part of this marked increase can be attributed to off-label use to treat behaviour disorders, attention deficit, hyperactivity, and anxiety disorders [17, 18].

There are still important gaps in evidence regarding gender differences and the treatment of mental disorders and in this vulnerable population under 17 years old [11, 17]. The profile of children and adolescent users of antipsychotics with early-onset schizophrenia is still controversial, and little is known about their treatment patterns in the real world, thus indicating the need for further study. The aim of this study was to investigate potential gender differences in the use of atypical antipsychotics among patients with early-onset schizophrenia.

## Method

### Study design

This was a cross-sectional study using records from the national ambulatory administrative database, Ambulatory Information System, SIA/SUS.

### Setting

The national administrative database Ambulatory Information System (SIA) of the Unified Health System (SUS), contains information about all procedures for outpatient care and dispensing of high-cost medicines for certain diseases according to Brazilian guidelines. This database is managed by the Ministry of Health and registers over 200 million procedures/month [19]. It has unrestricted access, and the data have been publicly available since 2008.

Atypical antipsychotics are considered high-cost medicines in Brazil, and the following medications are provided by SUS: oral clozapine, olanzapine, quetiapine, risperidone and ziprasidone. Injectable atypical antipsychotics are not available. These drugs are dispensed to patients only after analysing the request and determining whether the request is in compliance with Brazilian guidelines for the treatment of schizophrenia in adults. Then, atypical antipsychotics are provided to patients monthly for a period of 3 months, and the quantity prescribed could cover only a maximum duration of 90 days. After this period, a new request and analysis are necessary according to the rules in Brazil.

There are no specific national guidelines for the treatment of schizophrenia in children and adolescents. Clinicians use the guidelines for adults as a reference. Brazilian guidelines for the treatment of schizophrenia in adults recommend only atypical antipsychotic monotherapy and the use of clozapine only in cases in which patients are refractory to at least two other antipsychotics [20]. For this reason, no patient was identified using more than one antipsychotic in this database.

### Participants

We included all patients aged 10 to 17 years old who were diagnosed with schizophrenia according to the International Classification of Diseases, tenth Revision, Clinical Modification (ICD-10) and received at least one prescription of atypical antipsychotics through a pharmaceutical assistance programme from the SUS between 2008 and 2017.

### Variables

We compared gender differences in the demographic and clinical characteristics of the study participants, and they were classified by gender for further analysis. The baseline demographic variables considered were sex, age at study entry, race, geographic region of residence at study entry, and year of study entry (defined as the year of the first provision of atypical antipsychotics from January 1, 2008, to December 31, 2017). The baseline clinical variables considered were diagnosis according to ICD-10 at study entry (paranoid schizophrenia or other types) and type of atypical antipsychotic used at study entry.

### Measurement

The mean dose of the antipsychotics used was investigated according to the age group (10–12 years and 13–17 years) and compared by gender differences. We calculated the mean dose and defined daily dose (DDD) ratios to make comparisons between five types of antipsychotics available in the SUS. The DDD is a standardized unit of measurement in pharmacological studies, defined by the average maintenance dose of a drug in adults and allows the comparison of drug use/consumption from different countries or between population groups [21]. The DDDs of the investigated oral antipsychotics are clozapine (300.0 mg), olanzapine (10.0 mg), risperidone (5.0 mg), quetiapine (400.0 mg), and ziprasidone (80.0 mg) [21].

### Statistical analysis

Initially, the characteristics of the patients were described and stratified by gender. Then, we calculated the Pearson chi-squared tests or independent sample t-tests to investigate the differences in demographic and clinical characteristics between male and female groups. The doses were described by means and standard deviations. The ratio mean dose/defined daily dose (DDD) was calculated to normalize the doses and make comparisons. The rate of antipsychotic use per year was defined by percentage and plotted on a graph to show the trend of use over the years of the study. A significance level of *P* < 0.05 and a confidence interval of 95% were adopted.

## Results

**Fig. 1:**
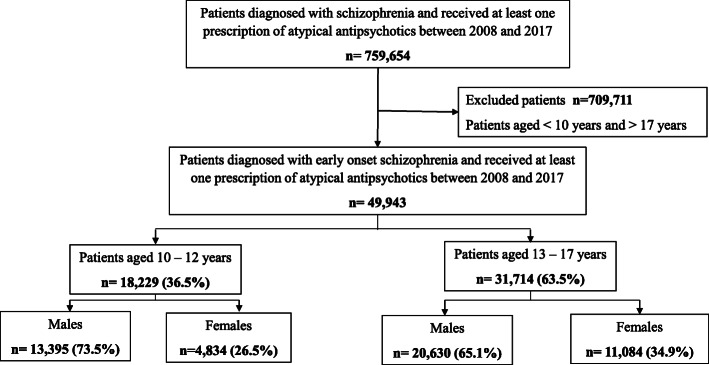
Flow chart of study

We identified 49,943 patients aged 10–17 years who were diagnosed with schizophrenia and who received at least one provision of atypical antipsychotics by the SUS from 2008 to 2017 (Fig. 1).

The most of patients were males (63.5%), with mean age 13.6 (±2.5) years old, lived in the southeast of Brazil (57.7%), diagnosed with paranoid schizophrenia (71.8%), and used at study entry mainly risperidone (62.5%), olanzapine (19.6%), quetiapine (12.4%), ziprasidone (3.3%) and clozapine (2.2%).

**Fig. 2:**
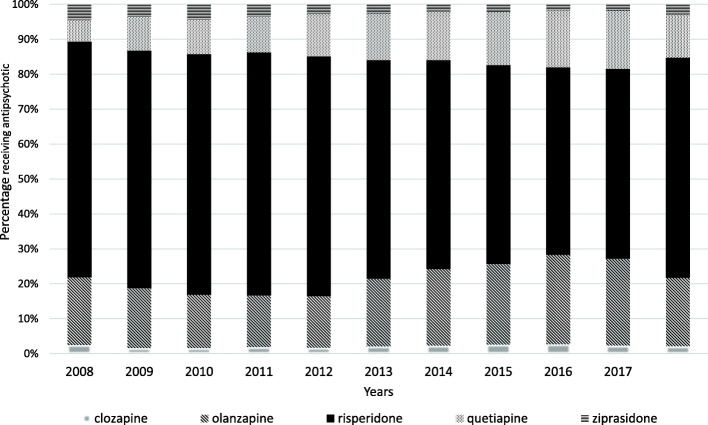
The percentage of children and adolescents receiving atypical antipsychotic over the period 2008–2017

Fig. 2 shows the trend of use of atypical antipsychotics over ten years of study. For the years 2008 to 2017, risperidone was the most prescribed atypical antipsychotic, reaching rates above 53%. Olanzapine was the second most common antipsychotic prescribed. The use trend of quetiapine and olanzapine increased especially from 2013.

The gender difference is highlighted especially in the group age of 13–17 years old (*p* < 0.001), residents in the southeast and south region (*p* < 0.001) of Brazil and who used risperidone at study entry (*p* < 0.001), with male predominance (Table 1). There was no gender difference in the use of clozapine.

**Table 1:** **Demographic and clinical characteristics of patients with early-onset schizophrenia by genders from 2008 to 2017 in Brazil**

Variables	Overall ***N*** = 49,943	Male ***N*** = 34,025	Female ***N*** = 15,918	P
**Demographic characteristics**				
Age at study entry (years)*				
10–12	18,229	13,395 (73.5)	4834 (26.5)	< 0.001
13 to 17 years	31,714	20,630 (65.1)	11,084 (34.9)	
**Mean age (SD)**^**&**^				
	13.6 (±2.48)	13.45 (±2.51)	13.91 (±2.40)	< 0.001
Race*				
White	6096	4137 (67.9)	1959 (32.1)	0.451
Black	579	404 (69.8)	175 (30.2)	
Pardo	4154	2795 (67.3)	1359 (32.7)	
Yellow	1073	733 (68.3)	340 (31.7)	
Indigenous	26	21 (80.7)	5 (19.3)	
No information	38,015	25,935 (68.2)	12,080 (31.8)	
Year of study entry*				
2008	8272	5710 (69.1)	2562 (30.9)	0.113
2009	4341	2899 (66.8)	1442 (33.2)	
2010	4654	3188 (68.5)	1466 (31.5)	
2011	4711	3198 (67.9)	1513 (32.1)	
2012	4156	2812 (67.6)	1344 (32.3)	
2013	4380	2929 (66.9)	1451 (33.1)	
2014	4582	3148 (68.7)	1434 (31.3)	
2015	5191	3575 (68.9)	1616 (31.1)	
2016	5108	3501 (68.5)	1607 (31.5)	
2017	4548	3065 (67.4)	1483 (32.6)	
Geographic region of residence at study entry*				
North	1319	840 (63.7)	479 (36.3)	< 0.001
Northeast	10,244	6858 (66.9)	3386 (33.1)	
Southeast	28,835	19,703 (68.3)	9132 (31.7)	
South	6645	4679 (70.4)	1966 (29.6)	
Midwest	2900	1945 (67.1)	955 (32.9)	
**Clinical characteristics**				
Diagnosis at study entry*				
Paranoid schizophrenia	35,887	24,394 (67.9)	11,493 (32.1)	0.240
Other types of schizophrenia	14,056	9631 (68.5)	4425 (31.5)	
Atypical antipsychotics used at study entry*				
Clozapine	1092	731 (66.9)	361 (33.1)	0.395
Olanzapine	9799	6511 (66.5)	3288 (33.5)	< 0.001
Risperidone	31,193	22,497 (72.1)	8696 (27.9)	< 0.001
Quetiapine	6183	3357 (54.3)	2826 (45.7)	< 0.001
Ziprasidone	1665	919 (55.2)	746 (44.8)	< 0.001

^&^ Statistic values were expressed by independent t test; * statistic values were expressed by Pearson’s chi-squared tests

The year at study entry did not differ significantly between sexes over 10 years. Race and type of schizophrenia also did not differ between genders.

Table 2 shows the doses prescribed to patients aged 10–12 years. There were no significant gender differences in the dosing of atypical antipsychotics prescribed in this age range. The ratio of mean dose/DDD was lower than 0.54, which is lower than DDD in adults.

**Table 2:** **Antipsychotic prescription and their mean dose for patients age 10–12 years with early-onset schizophrenia**

Variables	Overall ***N*** = 18,229	Male ***N*** = 13,395	Female ***N*** = 4834	P*
**Atypical antipsychotic**				
**Clozapine**				
Dose (mg/day)^&^	150.82 ± 138.79	144.49 ± 138.15	165.13 ± 140.31	0.334
Mean dose/DDD ratio	0.502 ± 0.46	0.481 ± 0.46	0.55 ± 0.46	
**Olanzapine**				
Dose (mg/day)^&^	5.18 ± 15.1	5.43 ± 17.72	4.56 ± 3.82	0.261
Mean dose /DDD ratio	0.512 ± 1.51	0.54 ± 1.77	0.45 ± 0.38	
**Risperidone**				
Dose (mg/day)^&^	1.19 ± 2.11	1.19 ± 1.83	1.21 ± 2.79	0.501
Mean dose /DDD ratio	0.24 ± 0.42	0.24 ± 0.36	0.24 ± 0.55	
**Quetiapine**				
Dose (mg/day)^&^	81.22 ± 109.02	85.06 ± 116.13	74.37 ± 94.78	0.077
Mean dose /DDD ratio	0.20 ± 0.27	0.21 ± 0.29	0.18 ± 0.23	
**Ziprasidone**				
Dose (mg/day)^&^	43.43 ± 33.61	43.11 ± 33.36	43.94 ± 34.10	0.805
Mean dose /DDD ratio	0.54 ± 0.42	0.53 ± 0.42	0.54 ± 0.42	

^&^ t-test was performed to compare mean dose (mg/day) between male and female; * *p*-value refers to the difference between male and female

Table 3 shows the doses prescribed to patients aged 13–17 years. There were gender differences in the dosing of olanzapine (5.60 mg/day for males, 5.09 mg/day for females, *p* = 0.012) and quetiapine (114.87 mg/day for males, 100.28 mg/day for females, *p* = 0.041), such that males received slightly higher doses of olanzapine and quetiapine than females aged 13 to 17 years old. Considering the rate mean dose/DDD, we observed that prescribed doses were lower than DDD, which is lower than 0.76 in adults.

**Table 3:** Antipsychotic prescription and their mean dose for patients age 13–17 years with early-onset schizophrenia

Variables	Overall ***N*** = 31,714	Male ***N*** = 20,630	Female ***N*** = 11,084	P*
**Atypical antipsychotic**				
C**lozapine**				
Dose (mg/day)^&^	184.45 ± 338.72	178.88 ± 166.22	195.43 ± 535.82	0.490
Mean dose/DDD ratio	0.61 ± 1.12	0.59 ± 0.55	0.65 ± 1.78	
**Olanzapine**				
Dose (mg/day)^&^	5.42 ± 8.57	5.60 ± 9.85	5.09 ± 5.35	0.012
Mean dose /DDD ratio	0.54 ± 0.85	0.56 ± 0.98	0.51 ± 0.53	
**Risperidone**				
Dose (mg/day)^&^	1.42 ± 2.66	1.44 ± 2.40	1.37 ± 3.18	0.093
Mean dose /DDD ratio	0.28 ± 0.53	0.29 ± 0.48	0.27 ± 0.63	
**Quetiapine**				
Dose (mg/day)^&^	107.78 ± 247.59	114.87 ± 327.43	100.28 ± 112.41	0.041
Mean dose /DDD ratio	0.26 ± 0.62	0.28 ± 0.82	0.25 ± 0.28	
**Ziprasidone**				
Dose (mg/day)^&^	61.10 ± 131.82	65.41 ± 148.99	56.22 ± 109.02	0.220
Mean dose /DDD ratio	0.76 ± 1.64	0.81 ± 1.86	0.70 ± 1.36	

^&^ t-test was performed to compare mean dose (mg/day) between male and female; * *p*-value refers to the difference between male and female

## Discussion

### Main findings

Our findings highlighted the outpatient use of atypical antipsychotics by gender in a large and representative population of children and adolescents assisted by the public health system in Brazil. The most commonly prescribed atypical antipsychotic among children and adolescents was risperidone, followed by olanzapine. Gender differences were observed, especially in the 12–17 years age group and in the choice of atypical antipsychotics prescribed, such as risperidone and olanzapine, with significant male predominance. The prescribed doses of olanzapine and quetiapine were slightly higher among males aged 13–17 years than among females.

### Comparison with previous studies

The onset of mental disorders, including schizophrenia and other psychotic disorders, commonly occurs in adolescence due to important changes in the brain structure with complex interactions among biological, psychological and social factors [10]. Most of the antipsychotic users identified in this study with a diagnosis of schizophrenia were adolescents in the age range 13–17 years, consistent with other studies from Canada, Taiwan, and Denmark [7, 8, 22].

There were more males than females receiving atypical antipsychotics in this study, similar to previous findings that also revealed gender differences with male predominance [6, 23, 24]. The fact that schizophrenia, like other mental disorders, is involved in complex biological (hormonal) and psychosocial interactions seems to cause gender differences. For many years, several studies have reported gender differences in the age at onset for schizophrenia [25]. Males have an earlier age at onset for schizophrenia (before 25 years) than females (after 25 years) [26]. An increasing number of studies are examining this topic and exploring the association between gender differences and the aetiology of psychosis disorders. Oestrogen seems to be psychoprotective and to influence the development and functioning of the brain in females, whereas hypothalamic–pituitary–gonadal dysfunction can influence both sexes [27]. On the other hand, some recent studies argue that the usual male predominance found in schizophrenia has become less apparent or that there is no gender difference [4, 7, 8]. Evidence regarding the gender difference in the risk of schizophrenia is still inconclusive [23].

While there is a significant increase in the use of atypical antipsychotics in children and adolescents worldwide, there are important gaps involving effectiveness, safety and effects in long-term use [11, 28]. Approximately 36% of patients aged 12 or below were using some of these atypical antipsychotics (risperidone, clozapine, olanzapine, quetiapine or ziprasidone), and none of these medications are approved for use in this age range (under 13 years) for the treatment of schizophrenia according to the National Sanitary Surveillance Agency (ANVISA-Brazil), Food and Drug Administration (FDA-US), or Medicines & Healthcare products regulatory Agency (MHRA-UK) [15, 16, 29].

Olanzapine, risperidone and quetiapine are approved for the treatment of schizophrenia only from 13 years old according to the ANVISA and FDA, while clozapine and ziprasidone are not approved for use among children and adolescents [16, 29]. However, the use of these drugs is increasing worldwide and exposes patients to potentially unnecessary harm.

Two systematic reviews assessed the effects of the use of antipsychotics in children (*n* = 6 RCTs, 256 participants before 13 years) [11] and adolescents (*n* = 13 RCTs, 1112 patients 13–18 years) [28] diagnosed with schizophrenia and identified benefits in using antipsychotics, but neither could conclude that any one antipsychotic is better than another, except clozapine. Clozapine is a good option for children and adolescents with treatment-resistant schizophrenia [9, 11, 14]. However, children and adolescents were more likely to show adverse effects; for instance, olanzapine, risperidone, and clozapine were often associated with weight gain in adolescents [12, 28]. Children who received clozapine showed a higher rate of neutropenia [11].

The high prescription rate of risperidone followed by olanzapine for the treatment of children and adolescents with schizophrenia has been confirmed in other studies [7, 17, 30]. This can be attributed to the increase in the diagnosis of schizophrenia and other mental disorders in this age group, the implementation of diagnostic criteria for schizophrenia, improved access to intervention services and an increase in treatment capacity [4, 8].

In addition, the diagnosis is complex, and the psychotic experience may be misdiagnosed as early-onset schizophrenia or be the manifestations of other mental disorders, such as behavioural disorders, anxiety disorders, affective disorders and developmental disorders [1, 14]. The use of off-label atypical antipsychotics to treat these disorders is also a common practice and can inflate the consumption of these medicines [17, 30, 31]. To verify this hypothesis, further and extensive research should be conducted.

This scenario in the real world is a concern, given the expansion in the profile of users of antipsychotics and the diagnoses for which they are used. The majority of antipsychotic use is off-label, and the effects on the developing brain of early and prolonged exposure to atypical antipsychotics are unknown [31, 32].

For instance, among antipsychotics, risperidone is often used for the treatment of disruptive behaviour disorders, including aggression and conduct disorders, attention-deficit-hyperactivity disorder (ADHD) and autism, despite little evidence regarding its effectiveness [33]. Evidence has shown that risperidone reduces aggression and conduct problems compared to placebo in patients aged 5 to 18 years old [33]. The effectiveness of risperidone in the treatment of ADHD is unclear [34], and autism shows some benefits in irritability, repetition and social withdrawal [35].

The prescribed doses are in compliance with the Brazilian clinical protocol, although there is no specific protocol for treatment in children and adolescents in the country [20]. The doses also corroborate the dosage recommended by British National Formulary for children, which recommends starting with low doses and gradually titrating upwards over the weeks [15].

Although no guidelines recommend gender as a factor in the choice of antipsychotic or dose, patient gender should be considered by clinicians. Data are conflicting, and clinical implications exist, suggesting that females have a better response to antipsychotic treatment and require lower doses than males [36–38]. We found a slight sex difference only in the dose of quetiapine and risperidone for males in the 13–17 year age group. Another study conducted in Taiwan found no gender difference in the prescribed doses of atypical antipsychotics [7]. The clinical response to antipsychotic treatment seems to vary between males and females, and more studies are needed.

### Strengths and limitations

To our knowledge, this is the first study to investigate gender differences in children and adolescents who received atypical antipsychotics with a diagnosis of schizophrenia in Brazil. The data were double checked, which increases the reliability of the findings. The database used represents the users of the public health system that covers more than 75% of the Brazilian population. There are few studies that report a large and representative population of children and adolescents who received antipsychotics in real words.

There are some limitations. The database is reliable and representative for studies of drug use, but they record only supply of the drugs, not whether the drugs were taken. We were unable to explore the concurrent use of other psychotropic drugs, adverse reactions, and the reasons that led clinicians to off-label use in children under 13 years of age. There was also no information on whether off-label use was a shared decision between clinicians and parents/caregivers.

## Conclusion

Despite limited evidence regarding the safety and efficacy of atypical antipsychotics in children and adolescents, this study revealed significant use of these drugs in this age group, and a large majority of these were boys. The findings support the gender difference, especially in the 13–17 year age group and in the choice of antipsychotic use, with greater emphasis on the use of risperidone and olanzapine.

The implementation of clinical protocols for the treatment of children and adolescents is needed and should be a key priority of mental health services in Brazil, since some antipsychotic prescriptions are off-label and the long-term effects are still known. The findings could also contribute to improving the use of atypical antipsychotics and the pharmacotherapy management of children and adolescents with schizophrenia in the real world. Furthermore, it suggests that gender differences should be considered in future research and clinical practice.

## Data Availability

The data generated and analysed during the current study are not publicly available but are available from the corresponding author on reasonable request.

## Abbreviations

ADHD: attention-deficit-hyperactivity disorder
ANVISA: National Sanitary Surveillance Agency, Brazil
DDD: Defined Daily Dose
EOS: Early-onset schizophrenia
FDA: Food and Drug Administration, US
ICD-10: International Classification of Diseases, tenth Revision
MHRA: Medicines & Healthcare products regulatory Agency, UK
RCTs: randomized controlled trials
SIA: Ambulatory Information System
SUS: Unified Health System
